# Supplementary material for: Removing Acrylic Conformal Coating with Safer Solvents for Re-Manufacturing Electronics
Source: Polymers (Basel). 2021 Mar 18;13(6):937. doi: 10.3390/polym13060937 (PMC8002995; doi:10.3390/polym13060937)
Supplement: Supplementary file 1 [file polymers-13-00937-s001.pdf]

Supporting Information

Section 1 Components of DCM based coating strippers

Table 1. Composition of Klean Strip Premium.

CAS #	Components	Concentration
75-09-2	Dichloromethane	70.0-95.0%
67-56-1	Methanol	< 5.0%
127087-87-0	Poly(oxy-1,2-ethandiyl)	< 5.0%
124-38-9	Carbon dioxide	< 5.0%

Table 2. Composition of Klean Strip X.

CAS #	Components	Concentration
75-09-2	Dichloromethane	30.0 – 40.0%
67-56-1	Methanol	15.0 – 26.0%
67-64-1	Acetone	< 10.0%
1330-20-7	Xylene	< 10.0%
108-88-3	Toluene	< 10.0%
100-41-4	Ethylbenzene	< 5.0%
64-17-5	Ethyl alcohol	< 5.0%
67-63-0	Isopropyl alcohol	< 5.0%

Section 2 Sample preparation for the dwell time test

As Figure S1 shows, a gasket was pasted on the conformal coating surface and a sheet of parafilm attached to the back of the printed circuit board to avoid solvent leakage during the dwell test.

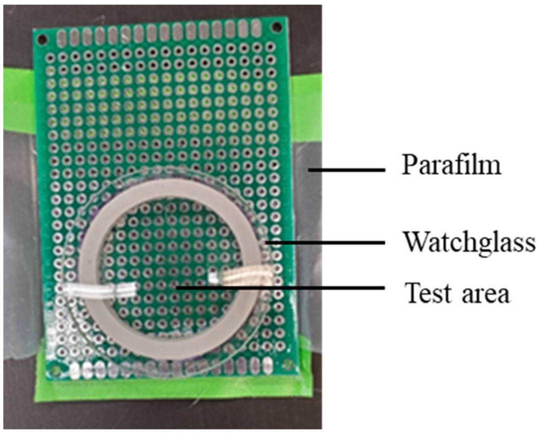

Figure 1. Sample preparation for the dwell time test.

Section 3 Thickness measurement of coated PCBs

Materials and Equipment

Printed circuit boards (PCB), acrylic conformal coating, tape, Dektak stylus profiler (Bruker, Arizona, USA).

Methods

A piece of tape was attached on a PCB before coating. The coating was applied on the PCB using the same method as dip coating in the dwell time test. The PCB was stationed and dried at room temperature for over 24 hours. The tape was then torn out to create a coating step.

The PCB was fixed on the detection table using tapes. The stylus scanned from substrate to coated area. The scan duration was 10 seconds and scan length was 1500 microns. Three scan pathways were performed with the same scan direction.

Result of Thickness Measurement

The reference surface was set as close as possible to the start scanning point. The measured surface was placed close to end point. The coating height was measured from the differentials of coating height and substrate height. The measured coating thickness was 126 microns with standard deviation 4.2.

Section 4 Available GSK Solvent Safety Values

Table 3. GSK health score for solvents included in optimization.

Compound	CAS Number	Health Score
Dichloromethane *	75-09-2	4
Methyl Acetate	79-20-9	7
Cyclopentyl Methyl Ether	5614-37-9	4
Methyl Iso-Butyl Carbinol	108-11-2	N/A [b]
Propylene Glycol Monoethyl Ether Acetate	763-69-9	N/A [b]
p-Cymene	99-87-6	N/A [b]
Ethyl Acetate	141-78-6	8
Ektapro Eep	763-69-9	N/A [b]
d-Limonene	138-86-3	N/A [b]
Butyl Diglycol Acetate	124-17-4	N/A [b]
1,3-Dioxolane	646-06-0	N/A [b]
Methyl Oleate	112-62-9	N/A [b]
Cyclohexane	110-82-7	7
Dibasic Esters (Dbe)	106-65-0	N/A [b]
Benzyl Alcohol	100-51-6	7
Di-Isobutyl Ketone	108-83-8	N/A [b]
Anisole	100-66-3	7
Propylene Glycol Monobutyl Ether	9038-95-3	N/A [b]
Texanol	25265-77-4	N/A [b]
Methyl Cyclohexane	108-87-2	8
Iso-Pentyl Alcohol	123-51-3	7
n-Butyl Propionate	590-01-2	N/A [b]
Ethylene Glycol Monobutyl Ether	111-76-2	N/A [b]
Glycerol Triacetate	102-76-1	N/A [b]
n-Butyl Acetate	123-86-4	8
Butyl Benzoate	136-60-7	N/A [b]
Iso-Butyl Isobutyrate	97-85-8	N/A [b]
Dipropylene Glycol Methyl Ether	34590-94-8	N/A [b]
n-Propyl Acetate	109-60-4	8
n-Amyl Alcohol	71-41-0	N/A [b]
Propylene Glycol Monomethyl Ether Acetate	108-65-6	N/A [b]
Diacetone Alcohol	123-42-2	N/A [b]
Dipropylene Glycol Mono N-Butyl Ether	29911-28-2	N/A [b]
Propylene Glycol Monomethyl Ether	107-98-2	N/A [b]
Iso-Pentyl Acetate	204-633-5	N/A [b]
Iso-Propyl Acetate	108-21-4	7
Acetone	67-64-1	8

n-Amyl Acetate	628-63-7	N/A [b]
t-Butyl Acetate	540-88-5	8
Dimethyl Isosorbide	31692-85-0	N/A [b]
Methyl Iso-Amyl Ketone	110-12-3	N/A [b]
Propylene Glycol Phenyl Ether	770-35-4	N/A [b]
n-Propyl Propanoate	539-82-2	N/A [b]
Ethyl Lactate	687-47-8	N/A [b]
Diethylene Glycol Monobutyl Ether	112-34-5	7
2-Phenoxy Ethanol	2807-30-9	N/A [b]
1-Nitropropane	108-03-2	N/A [b]
Methyl Ethyl Ketone (Mek)	78-93-3	8
Cyclohexanone	108-94-1	6
Methyl Propyl Ketone	107-87-9	6
Hexylene Glycol	107-41-5	N/A [b]
Glycerol Diacetate	102-62-5	N/A [b]
1-Butanol	71-36-3	5
2-Propanol	67-63-0	8
2-Butanol	78-92-2	8
Cyrene	53716-82-8	N/A [b]
Iso-Butanol	78-83-1	N/A [b]
t-Butyl Alcohol	75-65-0	6
1-Propanol	71-23-8	5
Caprolactone (Epsilon)	502-44-3	N/A [b]

[*]Dichloromethane was not used in the optimization but is included in this table as a benchmark to which other solvents can be compared; [b]Not available in the GSK database.

Section 5 Final Optimized Solvent List

Table 4. Final optimized solvent list with GSK score.

Solvent A	Solvent B	Volume Fraction A (%)	Volume Fraction B (%)	HSP Distance (MPa$^{1/2}$)	δD	δP	δH	GSK Health Score (A)	GSK Health Score (B)
Butyl Diglycol Acetate	Propylene Glycol Monoethyl Ether Acetate	89	11	3.089	16	4.3	8.1	N/A [a]	N/A [a]
Butyl Diglycol Acetate	Methyl Cyclohexane	69	31	1.781	16	2.8	6	N/A [a]	8
Butyl Diglycol Acetate	n-Propyl Acetate	87	13	3.094	15.9	4.1	8.1	N/A [a]	8
Butyl Diglycol Acetate	Cyclohexanone	69	31	2.371	16.6	5.4	7.2	N/A [a]	6
Acetone	Methyl Cyclohexane	46	54	2.242	15.8	4.8	3.8	8	8
Butyl Diglycol Acetate	Cyclohexane	70	30	1.448	16.2	2.9	5.8	N/A [a]	7
Diacetone Alcohol	Methyl Cyclohexane	46	54	1.288	15.9	3.8	5.5	N/A [a]	8
Diethylene Glycol Monobutyl Ether	Methyl Cyclohexane	49	51	1.308	16	3.4	5.7	7	8
Ethylene Glycol Monobutyl Ether	Methyl Cyclohexane	45	55	2.233	16	2.3	6.1	N/A [a]	8
Dibasic Esters (Dbe)	Propylene Glycol Monobutyl Ether	85	15	3.967	16.1	6.2	8.5	N/A [a]	N/A [a]
Butyl Diglycol Acetate	p-Cymene	56	44	0.894	16.6	3.3	5.6	N/A [a]	N/A [a]
Butyl Diglycol Acetate	1-Nitropropane	88	12	2.917	16.1	5.1	7.9	N/A [a]	N/A [a]
Butyl Benzoate	Butyl Diglycol Acetate	41	59	2.093	16.9	4.7	7.1	N/A [a]	N/A [a]
Cyclohexanone	Ethyl Acetate	20	80	2.444	16.2	5.9	6.8	6	8
Cyclohexanone	Dibasic Esters (Dbe)	31	69	3.668	16.7	7.1	7.4	6	N/A [a]
Ethyl Acetate	Methyl Cyclohexane	72	28	1.37	15.9	3.8	5.5	8	8
Dibasic Esters (Dbe)	Methyl Cyclohexane	60	40	0.84	16.1	3.9	5.4	N/A [a]	8
Methyl Cyclohexane	Methyl Iso-Butyl Carbinol	57	43	3.153	15.7	1.4	5.9	8	N/A [a]
Methyl Cyclohexane	Texanol	48	52	2.203	15.5	3.2	5.6	8	N/A [a]
Cyclohexanone	Methyl Iso-Butyl Carbinol	65	35	3.546	17	6.6	7.6	6	N/A [a]
Methyl Cyclohexane	Propylene Glycol Monomethyl Ether	54	46	1.945	15.8	2.9	5.9	8	N/A [a]
Cyclohexanone	Texanol	57	43	3.796	16.6	7.4	7.1	6	N/A [a]
Di-Isobutyl Ketone	Butyl Diglycol Acetate	71	29	1.078	16	3.8	5.3	N/A [a]	N/A [a]
Butyl Diglycol Acetate	Methyl Propyl Ketone	58	42	2.312	16	5.6	6.7	N/A [a]	6
Ethylene Glycol Monobutyl Ether	Methyl Oleate	10	90	0.701	16.2	3.9	5.3	N/A [a]	N/A [a]
Ethyl Acetate	Methyl Oleate	20	80	0.835	16.1	4.1	5	8	N/A [a]
Cyclohexanone	Methyl Cyclohexane	56	44	2.292	17	4.7	3.3	6	8
Methyl Acetate	Methyl Cyclohexane	59	41	1.682	15.7	4.2	4.9	7	8
Methyl Oleate	Propylene Glycol Monomethyl Ether	90	10	0.765	16.1	4.1	5.2	N/A [a]	N/A [a]
Cyclohexanone	Methyl Acetate	40	60	3.821	16.4	7.7	6.6	6	7
Cyclohexane	Acetone	51	49	2.135	16.2	5.1	3.5	7	8
Ethyl Acetate	Methyl Propyl Ketone	78	22	2.572	15.8	5.8	6.7	8	6
Cyclohexanone	Methyl Oleate	12	88	0.794	16.4	4.4	4.6	6	N/A [a]
Cyclohexane	Ethyl Acetate	28	72	0.926	16.1	3.8	5.2	7	8
Methyl Acetate	Methyl Oleate	11	89	0.91	16.1	4.2	4.8	7	N/A [a]

Methyl Oleate	Propylene Glycol Mono-butyl Ether	89	11	0.906	16.1	3.9	5	N/A [a]	N/A [a]
Dipropylene Glycol Methyl Ether	Methyl Cyclohexane	48	52	2.133	15.8	2.7	5.9	N/A [a]	8
p-Cymene	Ethyl Acetate	41	59	0.14	16.5	4.1	5.2	N/A [a]	8
Cyclohexanone	Dipropylene Glycol Methyl Ether	63	37	3.993	16.9	7.4	7.4	6	N/A [a]
Butyl Benzoate	Ethyl Acetate	36	64	1.884	16.7	5.4	6.6	N/A [a]	8
p-Cymene	Acetone	66	34	1.691	16.8	5.1	4	N/A [a]	8
Di-Isobutyl Ketone	Ethyl Acetate	69	31	1.188	15.9	4.2	5.1	N/A [a]	8
Cyclohexane	Diethylene Glycol Mono-butyl Ether	48	52	0.627	16.4	3.6	5.6	7	7
Methyl Cyclohexane	Propylene Glycol Phenyl Ether	52	48	1.758	16.7	2.5	6	8	N/A [a]
Cyclohexane	Dibasic Esters (Dbe)	38	62	0.197	16.4	4	5.3	7	N/A [a]
Methyl Cyclohexane	Propylene Glycol Mono-butyl Ether	42	58	2.424	15.6	2.6	5.8	8	N/A [a]
Cyclohexane	Diacetone Alcohol	52	48	0.432	16.3	3.9	5.3	7	N/A [a]
Di-Isobutyl Ketone	Dibasic Esters (Dbe)	75	25	0.992	16.1	4.4	5.2	N/A [a]	N/A [a]
Cyclohexanone	Propylene Glycol Mono-butyl Ether	47	53	2.994	16.5	6.3	7.3	6	N/A [a]
Di-Isobutyl Ketone	Diethylene Glycol Mono-butyl Ether	83	17	1.055	16	4.3	5.2	N/A [a]	7
p-Cymene	Dibasic Esters (Dbe)	51	49	0.641	16.8	4.4	5.3	N/A [a]	N/A [a]
Dipropylene Glycol Mono N-Butyl Ether	Methyl Cyclohexane	52	48	1.583	15.8	3.4	5.7	N/A [a]	8
p-Cymene	Diethylene Glycol Mono-butyl Ether	62	38	0.735	16.9	4.1	5.5	N/A [a]	7
Cyclohexane	Ethylene Glycol Mono-butyl Ether	52	48	1.817	16.4	2.4	6	7	N/A [a]
Di-Isobutyl Ketone	Ethylene Glycol Mono-butyl Ether	85	15	1.058	16	3.9	5.3	N/A [a]	N/A [a]
Di-Isobutyl Ketone	Diacetone Alcohol	86	14	1.146	16	4.3	5	N/A [a]	N/A [a]
p-Cymene	Diacetone Alcohol	65	35	0.695	16.8	4.4	5.3	N/A [a]	N/A [a]
Methyl Oleate	n-Propyl Acetate	89	11	0.977	16.1	3.9	4.8	N/A [a]	8
p-Cymene	Ethylene Glycol Mono-butyl Ether	66	34	1.268	16.9	3.3	5.8	N/A [a]	N/A [a]
Ethyl acetate				2.683	15.8	5.3	7.2	8	
Butyl Diglycol Acetate				3.100	16	4.1	8.2	N/A [a]	
Propylene Glycol Monoethyl Ether Acetate				3.753	15.6	6.3	7.7	N/A [a]	

[a]Not available in the GSK database.
